# Educational

**Published:** 1869-10

**Authors:** 


### EDUCATIONAL.

Our Dental Colleges now soon begin their annual sessions; and we trust with such appreciation, sympathy and assistance by the whole profession as shall stimulate and make glad the hearts of those engaged in these enterprises of toil and anxiety. No one who has not been engaged can fully understand the feeling of responsibility and anxiety that attaches to the office of teacher, especially in the Dental profession; when so much is to be taught that is new, and for which there is little or no precedent— processes and modes that have not been settled by ages of experiment and demonstration.

Indeed, it has hitherto been the case, that so far as teaching the branches constituting Dentistry a specialty is concerned, every one has had to mark out a course for himself; or do what was about as difficult, select and collate from the entire field of Dental literature, and elsewhere too, whatever might bear upon and be made available in the particular branch taught.

The constantly recurring change in modes, processes and operations, adds still further difficulty to the work of Dental teaching. Every few months proposes, and very often effects some material and important change, in the application of principles, or methods of operation.

To keep up with the changes and improvements in the profession, requires constant energy, vigilance and industry, far more than one who merely sits down and receives what comes along, would imagine.

The work of the faithful Dental teacher is one of great toil and responsibility; and one who does not appreciate this, is not yet fully prepared for the work.

It is a matter of serious consideration whether the instruction that is given shall result in the increase or decrease of human suffering.

It is always a grave matter to give advice in things pertaining to human welfare, and a much more serious matter to educate and prepare those who are to deal with human suffering.

A Dentist through false teaching may be made a blunderer who goes along through a life time, making mistakes, the accumulated results of which are productive of untold misery to thousands, while he, who has been correctly taught—brought up in the right way—goes forth to his life work with an assurance of ability to mitigate pain and suffering, whenever found in his legitimate sphere. If these things are true, can it be sufficient to run at random over a general course, without fixing most permanently correct foundation principles?

Then let all engaged in teaching in our specialty see to it that nothing shall be wanting in the perfect fulfillment of a trust committed to their charge. T.

---

WE have received a communication from parties signing themselves, FORCEPS, ELEVATOR, ROSE PEARL BASE, and GOLD AND SILVER, relative to some parties practicing Dentistry in this city (Cincinnati) in violation of law.

Now, it is the business, and the duty of any and every one knowing of such violation, to report the same to the Prosecuting Attorney, or to the Grand Jury, giving the names of witnesses, and the matter will doubtless be attended to; or, if these parties fear to do this, let them give the names of the parties and witnesses, together with facts to some one who has interest and manhood enough to see that the law is executed. We doubt not it will be an easy matter to find many such. Each member of the Board of Examiners have just as much interest in the execution of the law, and responsibility in the prosecution of its violation, as anybody else, and no more. They were not appointed to institute prosecutions. But let us have the law executed promptly and fully. T.

